# WEPPcloud hydrologic and erosion simulation datasets from 28 watersheds in US Pacific Northwest and calibrating model parameters for undisturbed and disturbed forest management conditions

**DOI:** 10.1016/j.dib.2022.108251

**Published:** 2022-05-10

**Authors:** Mariana Dobre, Anurag Srivastava, Roger Lew, Chinmay Deval, Erin S. Brooks, William J. Elliot, Peter R. Robichaud

**Affiliations:** a Department of Soil and Water Systems, University of Idaho, Moscow, ID 83844-2060, USA; b Virtual Technology and Design, University of Idaho, Moscow, ID 83844-2481, USA; c USDA Forest Service, Rocky Mountain Research Station, 1221 South Main, Moscow, ID 83843, USA

**Keywords:** Hydrologic model outputs, Water Erosion Prediction Project (WEPP), Forested watersheds, Calibration, Decision-support tool, WEPPcloud

## Abstract

The WEPPcloud interface is a new online decision-support tool for the Water Erosion Prediction Project (WEPP) model that facilitates data preparation and model runs, and summarizes model outputs into tables and maps that are easily interpretable by users. The interface can be used by land and water managers in United States, Europe, and Australia interested in simulating streamflow, sediment and pollutant loads from both undisturbed and disturbed (e.g. post-wildfire or post-treatment such as thinning or prescribed fires) forested watersheds. This article contains full hydrologic model runs for 28 forested watersheds in the U.S. Pacific Northwest with the WEPPcloud online interface. It also includes links to repositories with the individual model runs, a table containing default model parameters for disturbed conditions, and figures with model outputs as compared to observed data. The data in the repositories include all the raw data input and output from the model as well as the processed data, which can be accessed through tables and shapefiles to provide additional insights into the model outputs. Lastly, the article describes how the data are organized and the content of each folder containing the data. These model runs are useful for anyone interested in modeling forested watersheds with the WEPPcloud interface.

## Specifications Table

**Table:** 

Subject	Hydrology and Water quality
Specific subject area	Decision-support tools in hydrology, soil erosion, and water quality
Type of data	Table Graphs Figures Model input and output GIS shapefiles
How the data were acquired	Data were acquired with WEPPcloud, a new decision-support tool developed to facilitate simulations of streamflow, sediment and phosphorus yield from forested watersheds.
Data format	Raw model input and output Analyzed model output data
Description of data collection	Both the raw input and output datasets were generated with the WEPPcloud (https://wepp.cloud/) interface and a modified version of the WEPP model. The raw input data were processed via WEPPcloud from a series of free primary national databases.
Data source location	All modeled watersheds are located in the United States: Lake Tahoe, California/Nevada: 39.0968° N, 120.0324° W Bull Run Watershed, Oregon: 45.4812° N, 121.9567° W Cedar River, Washington: 47.3431° N, 121.6086° W Mica Creek, Idaho: 47.1695° N, 116.2525° W The primary datasets used in WEPPcloud were accessed from: Topography: 10- and 30-m National Elevation Dataset (NED) https://www.usgs.gov/core-science-systems/national-geospatial-program/national-map Soils: SSURGO/STATSGO https://www.nrcs.usda.gov/wps/portal/nrcs/detail/soils/survey/?cid=nrcs142p2_053627 Climate: PRISM http://prism.oregonstate.edu Climate: Daymet https://daymet.ornl.gov Climate: gridMET http://www.climatologylab.org/gridmet.html Landuse: 2016 National Land Cover Database https://www.usgs.gov/centers/eros/science/national-land-cover-database?qt-science_center_objects=0#qt-science_center_objects
Data accessibility	Repository name: Hydroshare Data identification number (DOI): Shared as part of the URLs. See below. Direct URLs to the datasets: WEPPcloud interface https://doi.org/10.4211/hs.47a190100b254a4993c11c2abced411c Lake Tahoe, California/Nevada Third Creek https://doi.org/10.4211/hs.3fa7ac7454ff441792177a4347be7958
	Glenbrook https://doi.org/10.4211/hs.979a22cdf76248aca0f098367c6c839f Logan House https://doi.org/10.4211/hs.b2d20dff60f94cea9fdd38840b0ebb6d General Creek https://doi.org/10.4211/hs.50be0bc4d59748f6b9d94d4563cde478 Blackwood Creek https://doi.org/10.4211/hs.12fce010911045f5b879730ad1f38388 Incline Creek https://doi.org/10.4211/hs.7b93d165af88413894a13a5c5fcb918c Incline 2 Creek https://doi.org/10.4211/hs.23a77c5d77e84c0e8712e33fdbb74a2c Incline 3 Creek https://doi.org/10.4211/hs.d16ccc1dc20b4092b595abf770de8423 Upper Truckee 1 https://doi.org/10.4211/hs.b2750f72c1e645449345cdcb55061c99 Upper Truckee 3 https://doi.org/10.4211/hs.92eb2b264332441c9a0d1bd5ab339e51 Upper Truckee 5 https://doi.org/10.4211/hs.17883240ce834ea8b547757ff372f651 Ward Creek https://doi.org/10.4211/hs.13360da0dcc642438a976d92b5a8c762 Ward Creek 3 https://doi.org/10.4211/hs.7df31ac48217470e857aeb6627753bc4 Ward Creek 7 https://doi.org/10.4211/hs.01df9b2f8c2f4002a5ca3e9994f8cabc Trout Creek 1 https://doi.org/10.4211/hs.431e9c2104474c1a851efc951a95e5c0 Trout Creek 2 https://doi.org/10.4211/hs.5b3e6368d3aa4e7d80eaea703baa70d2 Trout Creek 3 https://doi.org/10.4211/hs.30e00298b661412990a1f39a2a77b3c1 Bull Run Watershed, Oregon Blazed Alder https://doi.org/10.4211/hs.39c851332b4446d2a398f1fafbee97a7 Bull Run near Multnomah https://doi.org/10.4211/hs.f3fcc78029b34170a12da890d69dd34f Cedar Creek https://doi.org/10.4211/hs.8b7ef268c81a4e92b9f866431023233c Fir Creek https://doi.org/10.4211/hs.3a96ca9c9f0d4019b5da19cd88fc194c Little Sandy https://doi.org/10.4211/hs.30c1694ee6f645c488c1374a2afcc0ef North Fork https://doi.org/10.4211/hs.ac0cf7902a384658a3648c4130810ac8 South Fork https://doi.org/10.4211/hs.525c512ee899485baf2cede46ee24d6b Cedar River Watershed, Washington Upper Cedar River https://doi.org/10.4211/hs.592190aa103c474fac818b3d0c05db08 Taylor Creek https://doi.org/10.4211/hs.722979e575b2405c92e2f3d6937a12d8 Mica Creek, Idaho Watershed 3 https://doi.org/10.4211/hs.8c7dc32a87bc4c4cbd04c05262875d04 Watershed 6 https://doi.org/10.4211/hs.5758f9322b514671b870a3d339ef80c8
Related research article	Dobre, M., A. Srivastava, R. Lew, D. Chinmay, E.S. Brooks, W.J., Elliot, P.R. Robichaud (2022) WEPPcloud: An online watershed-scale hydrologic modeling tool. Part II. Model performance assessment and applications to forest management and wildfires. J. Hydrol. 127776. https://doi.org/10.1016/j.jhydrol.2022.127776.

## Value of the Data

• These datasets contain: 1) model simulation data from the WEPPcloud online interface. Specifically, they provide simulated daily streamflow and annual sediment and phosphorus yield for undisturbed forested conditions; 2) graphs of model data as compared to United States Geological Survey (USGS) data observed at the outlet of watersheds; and 3) a table with default model parameters.
• These datasets offer insight into the WEPPcloud's capability to simulate daily streamflow, and annual sediment and phosphorus yield from undisturbed forests with minimal calibration.
• Main beneficiaries of these resources are land and water managers and researchers interested in the accuracy of the WEPPcloud interface as well as anyone learning about the WEPP model and the WEPPcloud interface.
• Users can either recreate and run the watersheds in WEPPcloud or they can run the model with the provided files.

## 1 Data Description

These data were used in a WEPPcloud model assessment study: WEPPcloud: An online watershed-scale hydrologic modeling tool. Part II. Model performance assessment and applications to forest management and wildfires [1] and are also part of an additional study on the impacts of future forest management options on water quality in the Lake Tahoe basin, California/Nevada [2].

**Fig. 1:**
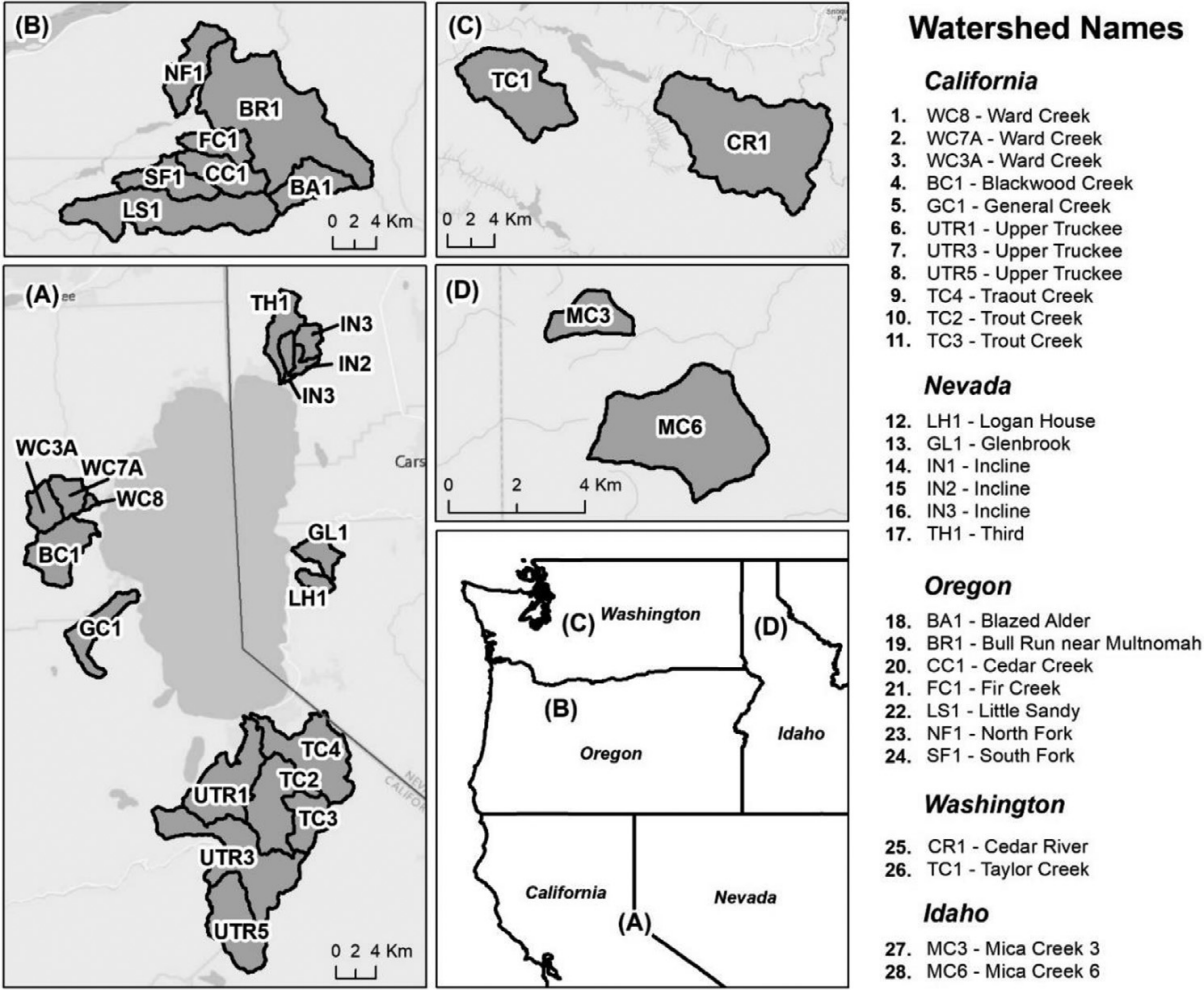
Location of the gauged study watersheds in the Western U.S.
- Table 1 contains information on modeled watersheds, including watershed name, USGS watershed name and station, and web links to model runs in WEPPcloud. The model runs are also archived in the HydroShare repository and contain both the input and the output data from the model, among other useful information. The watershed names reflect the watershed names used in other studies, which provided the observed water quality data for model assessment [3]. The streamflow for Mica Creek watersheds, MC3 and MC6, were recorded with flumes. Details regarding data collection can be found in [4].

- Fig. 1 shows the location of the modeled watersheds in the Western U.S.

**Table 1:** Watershed information and web links to model runs.

No.	Name	USGS station	USGS Name/Watershed Name Location
***California***			

1	WC8	10336676	WARD C AT HWY 89 NR TAHOE PINES https://wepp.cloud/weppcloud/runs/lt_202012_63_Ward_Creek_CurCond/cfg/
2	WC7A	10336675	WARD C A STANFORD ROCK TRAIL XING NR TAHOE CITY https://wepp.cloud/weppcloud/runs/lt_202012_63_Ward_Creek_WC3A_CurCond/cfg/
3	WC3A	10336674	WARD C BL CONFLUENCE NR TAHOE CITY https://wepp.cloud/weppcloud/runs/lt_202012_63_Ward_Creek_WC7A_CurCond/cfg/
4	BC1	10336660	BLACKWOOD C NR TAHOE CITY https://wepp.cloud/weppcloud/runs/lt_202012_62_Blackwood_Creek_CurCond/cfg/
5	GC1	10336645	GENERAL C NR MEEKS BAY https://wepp.cloud/weppcloud/runs/lt_202012_56_General_Creek_CurCond/cfg/
6	UTR1	10336610	UPPER TRUCKEE RV AT SOUTH LAKE TAHOE https://wepp.cloud/weppcloud/runs/lt_202012_44_Upper_Truckee_River_Big_Meadow_Creek_CurCond/cfg/
7	UTR3	103366092	UPPER TRUCKEE RV AT HWY 50 ABV MEYERS https://wepp.cloud/weppcloud/runs/lt_202012_44_Upper_Truckee_River_UT3_CurCond/cfg/
8	UTR5	10336580	UPPER TRUCKEE RV AT S UPPER TRUCKEE RD NR MEYERS https://wepp.cloud/weppcloud/runs/lt_202012_44_Upper_Truckee_River_UT5_CurCond/cfg/
9	TC4	10336780	TROUT CK NR TAHOE VALLEY https://wepp.cloud/weppcloud/runs/lt_202012_43_Trout_Creek_CurCond/cfg/
10	TC2	10336775	TROUT CK AT PIONEER TRAIL NR SOUTH LAKE TAHOE https://wepp.cloud/weppcloud/runs/lt_202012_43_Trout_Creek_TC2_CurCond/cfg/
11	TC3	10336770	TROUT CK AT USFS RD 12N01 NR MEYERS https://wepp.cloud/weppcloud/runs/lt_202012_43_Trout_Creek_TC3_CurCond/cfg/

***Nevada***			

12	LH1	10336740	LOGAN HOUSE CK NR GLENBROOK https://wepp.cloud/weppcloud/runs/lt_202012_31_Logan_House_Creek_CurCond/cfg/
13	GL1	10336730	GLENBROOK CK AT GLENBROOK https://wepp.cloud/weppcloud/runs/lt_202012_29_Glenbrook_Creek_CurCond/cfg/
14	IN1	10336700	INCLINE CK NR CRYSTAL BAY https://wepp.cloud/weppcloud/runs/lt_202012_19_Incline_Creek_CurCond/cfg/
15	IN2	103366995	INCLINE CK AT HWY 28 AT INCLINE VILLEGE https://wepp.cloud/weppcloud/runs/lt_202012_19_Incline_Creek_IN2_CurCond/cfg/
16	IN3	103366993	INCLINE CK ABV TYROL VILLAGE NR INCLINE VILLAGE https://wepp.cloud/weppcloud/runs/lt_202012_19_Incline_Creek_IN3_CurCond/cfg/
17	TH1	10336698	THIRD CK NR CRYSTAL BAY https://wepp.cloud/weppcloud/runs/lt_202012_18_Third_Creek_CurCond/cfg/

***Oregon***			

18	BA1	14138800	BLAZED ALDER CREEK NEAR RHODODENDRON https://wepp.cloud/weppcloud/runs/portland_BlazedAlder_CurCond.202009.cl532_gridmet.chn_cs50/cfg/
19	BR1	14138850	BULL RUN RIVER NEAR MULTNOMAH FALLS https://wepp.cloud/weppcloud/runs/portland_BRnearMultnoma_CurCond.202009.cl532_gridmet.chn_cs200/cfg/
20	CC1	14139700	CEDAR CREEK NEAR BRIGHTWOOD https://wepp.cloud/weppcloud/runs/portland_CedarCreek_CurCond.202009.cl532_gridmet.chn_cs150/cfg/
21	FC1	14138870	FIR CREEK NEAR BRIGHTWOOD https://wepp.cloud/weppcloud/runs/portland_FirCreek_CurCond.202009.cl532_gridmet.chn_cs150/cfg/
22	LS1	14141500	LITTLE SANDY RIVER NEAR BULL RUN https://wepp.cloud/weppcloud/runs/portland_LittleSandy_CurCond.202009.cl532_gridmet.chn_cs110/cfg/
23	NF1	14138900	NORTH FORK BULL RUN RIVER NEAR MULTNOMAH FALLS https://wepp.cloud/weppcloud/runs/portland_NorthFork_CurCond.202009.cl532_gridmet.chn_cs140/cfg/
24	SF1	14139800	SOUTH FORK BULL RUN RIVER NEAR BULL RUN https://wepp.cloud/weppcloud/runs/portland_SouthFork_CurCond.202009.cl532_gridmet.chn_cs160/cfg/

***Washington***			

25	CR1	12115000	CEDAR RIVER NEAR CEDAR FALLS https://wepp.cloud/weppcloud/runs/seattle_k_Cedar_River_CurCond.202009.cl532_gridmet.chn_cs200/cfg/
26	TC1	12117000	TAYLOR CREEK NEAR SELLECK https://wepp.cloud/weppcloud/runs/seattle_k_Taylor_Creek_CurCond.202009.cl532_gridmet.chn_cs100/cfg/

***Idaho***			

27	MC3§	-	MICA CREEK EXPERIMENTAL WATERSHED WS3 https://wepp.cloud/weppcloud/runs/occluded-bankroll/13/
28	MC6§	-	MICA CREEK EXPERIMENTAL WATERSHED WS6 https://wepp.cloud/weppcloud/runs/srivas42-legged-make-believe/0/

§ Streamflow recorded with flumes; there were no USGS gauging stations available for these watersheds.
- Table 2 contains key soils and management parameters used to parameterize WEPPcloud by management and three soil types (i.e. granitic, volcanic, alluvial), for the modeled watersheds. These values were summaries from various field studies conducted by the United States Department of Agriculture (USDA), Forest Service, Rocky Mountains Research Station and from published research papers.

**Table 2:** Key hillslope soils and management parameters used to parameterize the WEPPcloud interface by management and soil types for the modeled watersheds.

		Soils			Managements		
Soil Type	Management Name	Critical Shear (Pa)	Interrill Erodibility (kg s m^−4^)	Rill Erodibility (s m^−1^)	Canopy Cover (fraction)	Interrill Cover (fraction)	Rill Cover (fraction)
Granitic	Old Forest	4	250000	0.00015	0.9	1	1
Granitic	Young Forest	4	400000	0.0002	0.8	1	1
Granitic	Forest Thinning 96% cover	4	400000	0.00004	0.4	0.96	0.96
Granitic	Forest Thinning 93% cover	4	400000	0.00004	0.4	0.93	0.93
Granitic	Forest Thinning 85% cover	4	400000	0.00004	0.4	0.85	0.85
Granitic	Forest Prescribed Fire	4	1000000	0.0003	0.85	0.85	0.85
Granitic	Forest Low Severity Fire	4	1000000	0.0003	0.75	0.8	0.8
Granitic	Forest Moderate Severity Fire	4	1000000	0.0003	0.4	0.5	0.5
Granitic	Forest High Severity Fire	4	1800000	0.0005	0.2	0.3	0.3
Granitic	Shrubs	4	141100	0.0000873	0.7	0.9	0.9
Granitic	Shrub Prescribed Fire	4	170100	0.000149	0.7	0.75	0.75
Granitic	Shrub Low Severity Fire	4	170100	0.000149	0.5	0.7	0.7
Granitic	Shrub Moderate Severity Fire	4	170100	0.000149	0.3	0.5	0.5
Granitic	Shrub High Severity Fire	4	948600	0.0004343	0.05	0.3	0.3
Granitic	Bare Slope	4	300000	0.005	0.05	0.2	0.2
Granitic	Sod Grass	4	196700	0.0004446	0.4	0.6	0.6
Granitic	Bunch Grass	4	196700	0.0004446	0.6	0.8	0.8
Alluvial	Old Forest	1	300000	0.0001	0.9	1	1
Alluvial	Young Forest	1	500000	0.00015	0.8	1	1
Alluvial	Forest Thinning 96% cover	1	500000	0.00003	0.4	0.96	0.96
Alluvial	Forest Thinning 93% cover	1	500000	0.00003	0.4	0.93	0.93
Alluvial	Forest Thinning 85% cover	1	500000	0.00003	0.4	0.85	0.85
Alluvial	Forest Prescribed Fire	1	1500000	0.0002	0.85	0.85	0.85
Alluvial	Forest Low Severity Fire	1	1500000	0.0002	0.75	0.8	0.8
Alluvial	Forest Moderate Severity Fire	1	1500000	0.0002	0.4	0.5	0.5
Alluvial	Forest High Severity Fire	1	2000000	0.0004	0.2	0.3	0.3
Alluvial	Shrubs	1	141100	0.0000873	0.7	0.9	0.9
Alluvial	Shrub Prescribed Fire	1	170100	0.000149	0.7	0.75	0.75
Alluvial	Shrub Low Severity Fire	1	170100	0.000149	0.5	0.7	0.7
Alluvial	Shrub Moderate Severity Fire	1	170100	0.000149	0.3	0.5	0.5
Alluvial	Shrub High Severity Fire	1	948600	0.0004343	0.05	0.25	0.25
Alluvial	Bare Slope	1	750000	0.004	0.05	0.2	0.2
Alluvial	Sod Grass	1	196700	0.0004446	0.4	0.6	0.6
Alluvial	Bunch Grass	1	196700	0.0004446	0.6	0.8	0.8
Volcanic	Old Forest	1.5	300000	0.00005	0.9	1	1
Volcanic	Young Forest	1.5	600000	0.0001	0.8	1	1
Volcanic	Forest Thinning 96% cover	1.5	600000	0.00002	0.4	0.96	0.96
Volcanic	Forest Thinning 93% cover	1.5	600000	0.00002	0.4	0.93	0.93
Volcanic	Forest Thinning 85% cover	1.5	600000	0.00002	0.4	0.85	0.85
Volcanic	Forest Prescribed Fire	1.5	1000000	0.0002	0.85	0.85	0.85
Volcanic	Forest Low Severity Fire	1.5	1000000	0.0002	0.75	0.8	0.8
Volcanic	Forest Moderate Severity Fire	1.5	1000000	0.0002	0.4	0.5	0.5
Volcanic	Forest High Severity Fire	1.5	1500000	0.0003	0.2	0.3	0.3
Volcanic	Shrubs	1.5	134500	0.0000846	0.7	0.9	0.9
Volcanic	Shrub Prescribed Fire	1.5	162200	0.0001444	0.7	0.75	0.75
Volcanic	Shrub Low Severity Fire	1.5	162200	0.0001444	0.5	0.7	0.7
Volcanic	Shrub Moderate Severity Fire	1.5	162200	0.0001444	0.3	0.5	0.5
Volcanic	Shrub High Severity Fire	1.5	904400	0.0004209	0.05	0.3	0.3
Volcanic	Bare Slope	1.5	600000	0.003	0.05	0.2	0.2
Volcanic	Sod Grass	1.5	187600	0.0004309	0.4	0.6	0.6
Volcanic	Bunch Grass	1.5	187600	0.0004309	0.6	0.8	0.8
- Figs. 2–10 show daily streamflow and annual sediment and phosphorus yield model outputs as compared to observed data. Modeled streamflow was compared to data from the USGS gauging stations for watersheds in the Lake Tahoe basin, Bull Run, and Cedar River watersheds, and data measured with flumes in the Mica Creek Experimental Watersheds, Idaho. Modeled sediment and phosphorus yield was compared to flow-weighted annual observations processed by [3].
- Figs. 11–13 show interpolated estimated values of baseflow, deep seepage recession coefficients, critical shear, and phosphorus concentrations in runoff, lateral flow, and baseflow for Lake Tahoe basin watersheds in California/ Nevada. These values were manually interpolated based on the calibrated values at the 17 watersheds in Lake Tahoe with long-term USGS streamflow data.
- All the model runs including all the data input and output can be accessed from the web links provided in Table 1 and are also stored in public repositories (see Data Accessibility).
- **Model runs folder** contains a list and description of all the folders in these model runs, which are archived as .zip files. The data structure in these folders is similar for all WEPPcloud model runs.

*Reprinted from Journal of Hydrology, 127776, Mariana Dobre, Anurag Srivastava, Roger Lew, Chinmay Deval, Erin S. Brooks, William J. Elliot, Peter R. Robichaud, WEPPcloud: An online watershed-scale hydrologic modeling tool. Part II. Model performance assessment and applications to forest management and wildfires, Copyright (2022), with permission from Elsevier.*

**Model runs folder**

**climate** (folder) contains:

- the climate files generated by hillslope in .prn and .cli formats

- the watershed climate file

- the original daymet/gridmet data that were used to generate the .cli files

**dem (**folder**)** contains:

- the 10- or 30-m Digital Elevation Map (DEM) derived from the National Elevation Dataset

- topaz folder containing the watershed delineation and all the maps created during the watershed delineation

**export** (folder) contains channels and subcatchments files in GIS format containing topographic characteristics (such as slope, aspect, or length), input data (soil and management), and output information (runoff, lateral flow, baseflow, sediment, pollutant, etc.). The file also contains several GeoTIFF maps used in the model run.

**landuse** (folder) contains landuse map (e.g. ascii map with the 2016 National Land Cover Database (NLCD) for US Locale. The NLCD codes are translated into WEPP-equivalent management files based on the mapping for the configuration.

**observed** (folder) contains observed data (if) provided by the user

**soils** (folder) contains the soil files in WEPP format by mapunit key (mukey) and a ssurgo soils map in ascii format

**watershed** (folder) contains files with slope information for each channel and hillslope

**wepp** (folder) with sub-folders:

**- wepp/flowpaths** contains model input and output based on the flowpaths option, if selected. If the flowpath option is selected, the WEPP model will be run for each map pixel. This folder contains the runs folder with all the input data and an output folder with the runoff and soil loss for each flowpath.

**Fig. 2:**
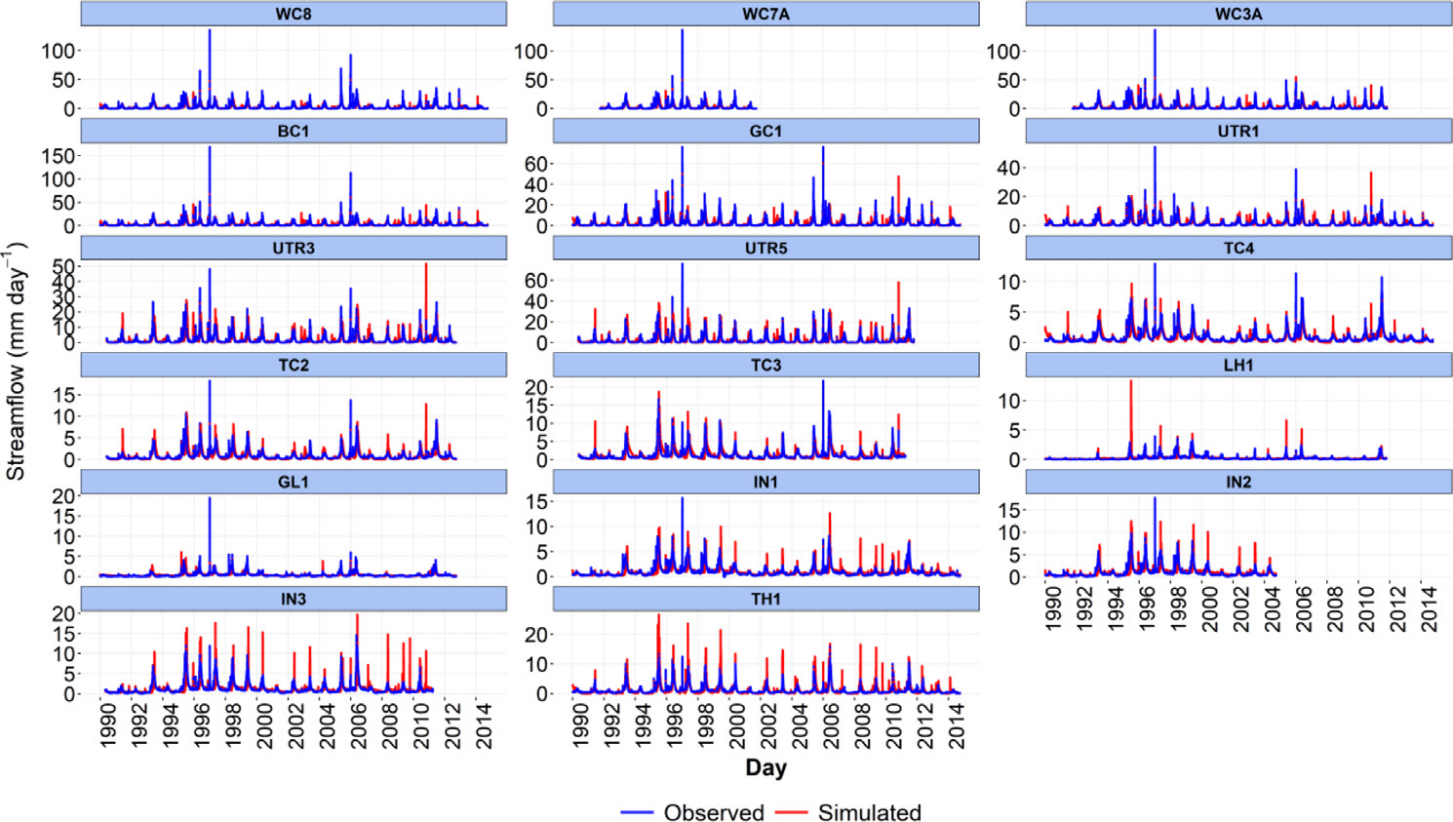
Simulated and observed daily streamflow at watersheds from the Lake Tahoe basin in California/Nevada.

**Fig. 3:**
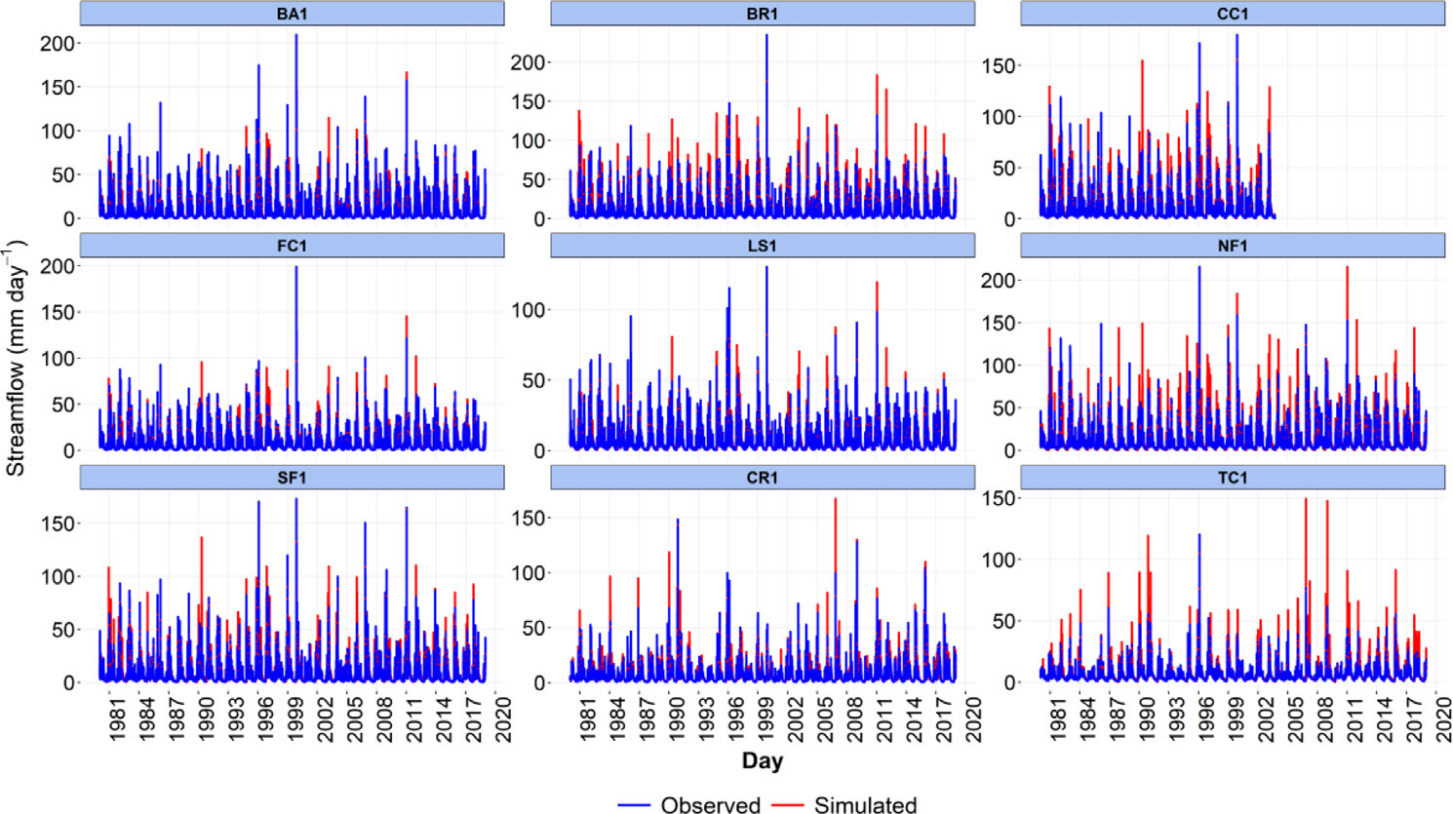
Simulated and observed daily streamflow from the Bull Run Watershed in Oregon and at Cedar River and Taylor Creek Watersheds in Washington.

**Fig. 4:**
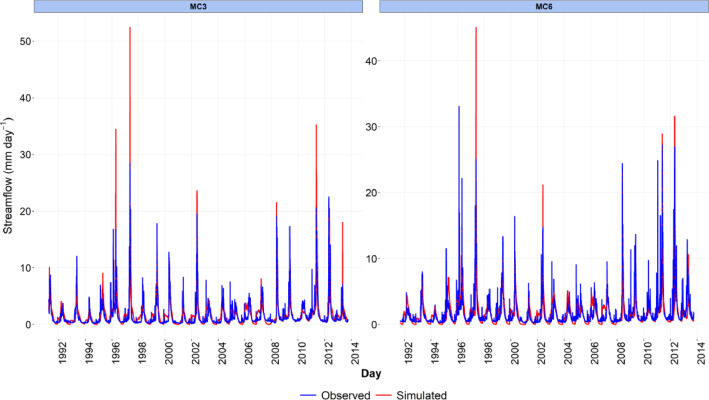
Simulated and observed daily streamflow at the Mica Creek Experimental Watersheds in Idaho.

**Fig. 5:**
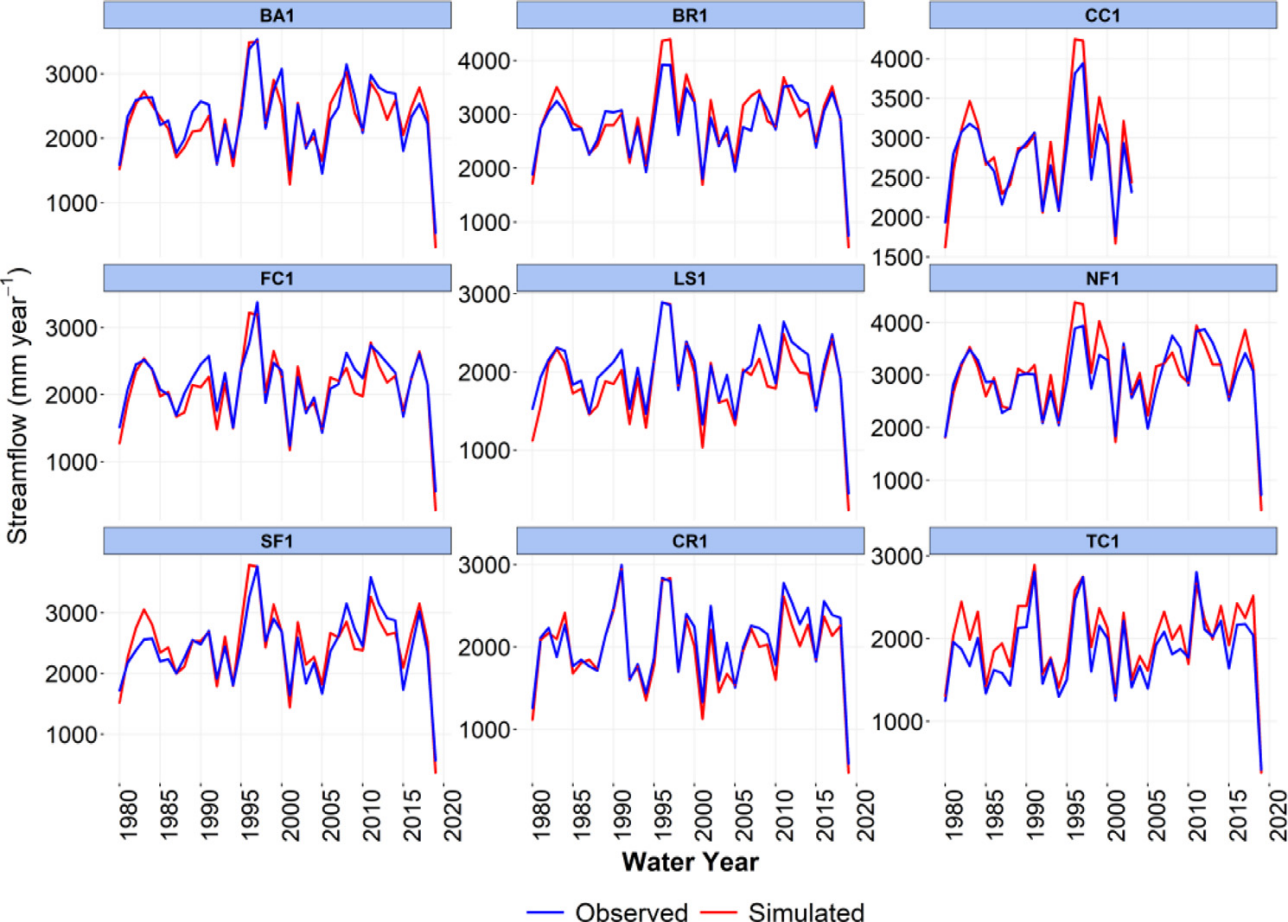
Simulated and observed total annual streamflow from the Bull Run Watershed in Oregon, Cedar River, and Taylor Creek Watersheds in Washington.

**Fig. 6:**
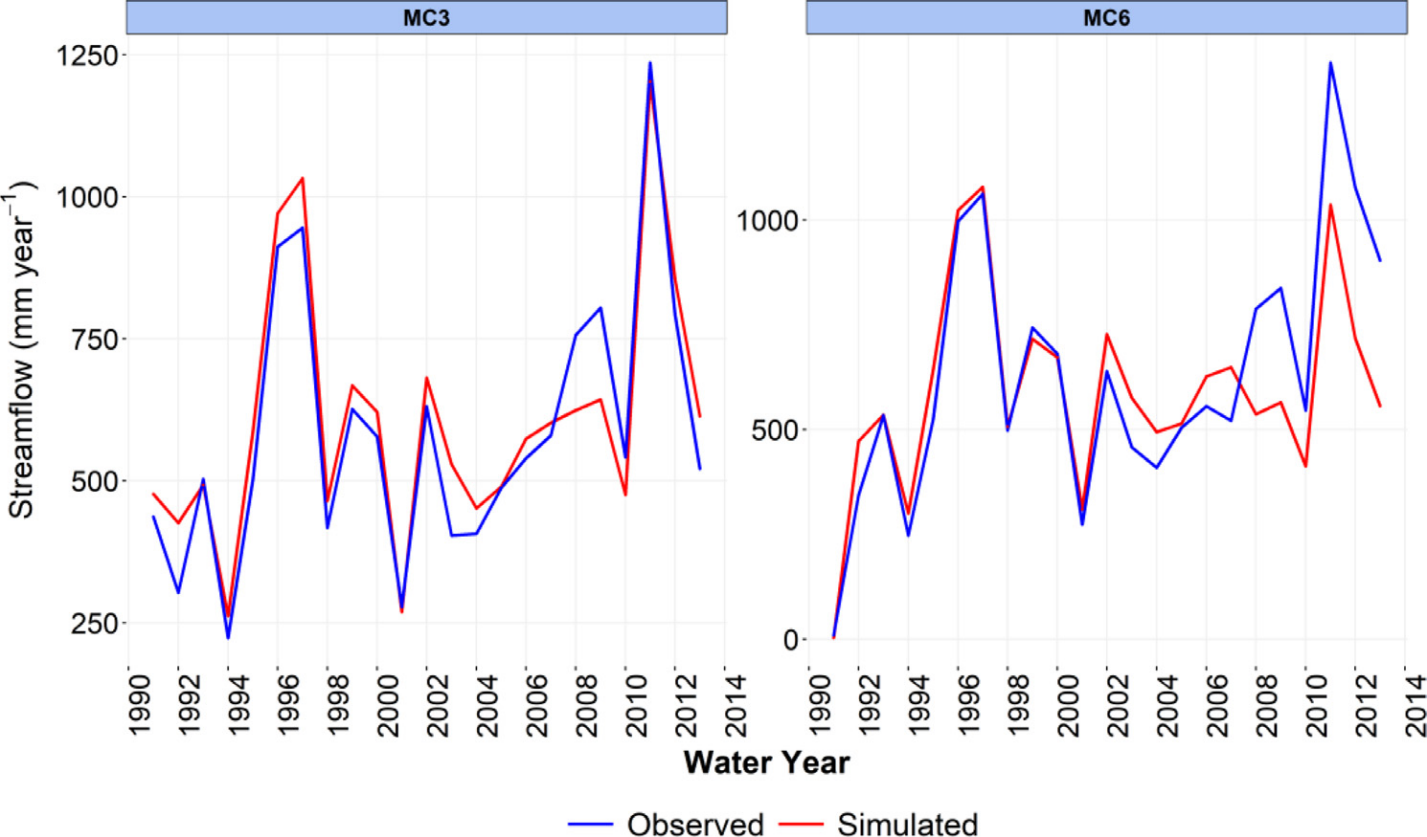
Simulated and observed total annual streamflow at watersheds from the Mica Creek Experimental Watershed in Idaho.

**Fig. 7:**
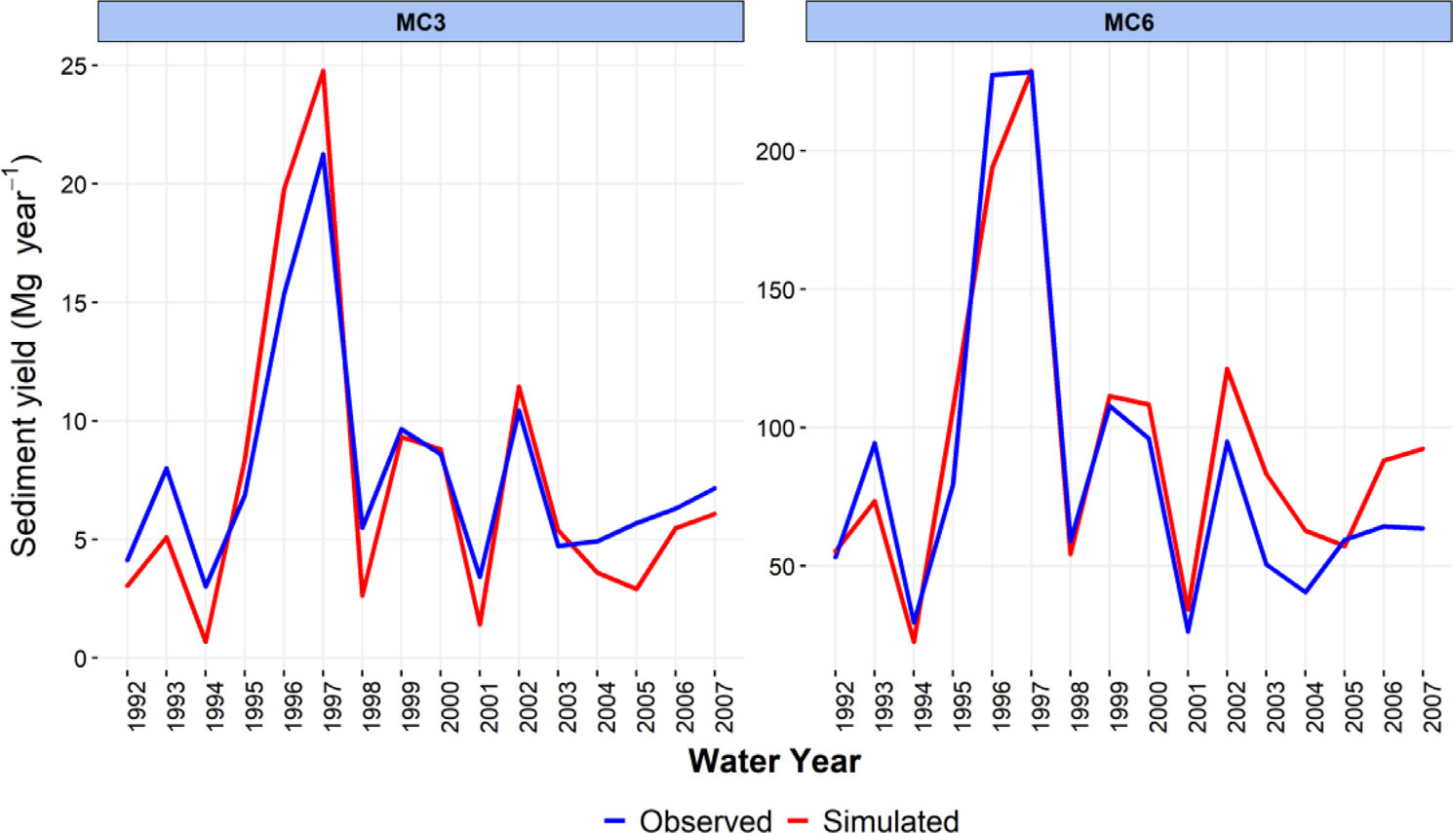
Simulated and observed total mean annual sediment load for watersheds in Mica Creek Experimental Watershed in Idaho.

**Fig. 8:**
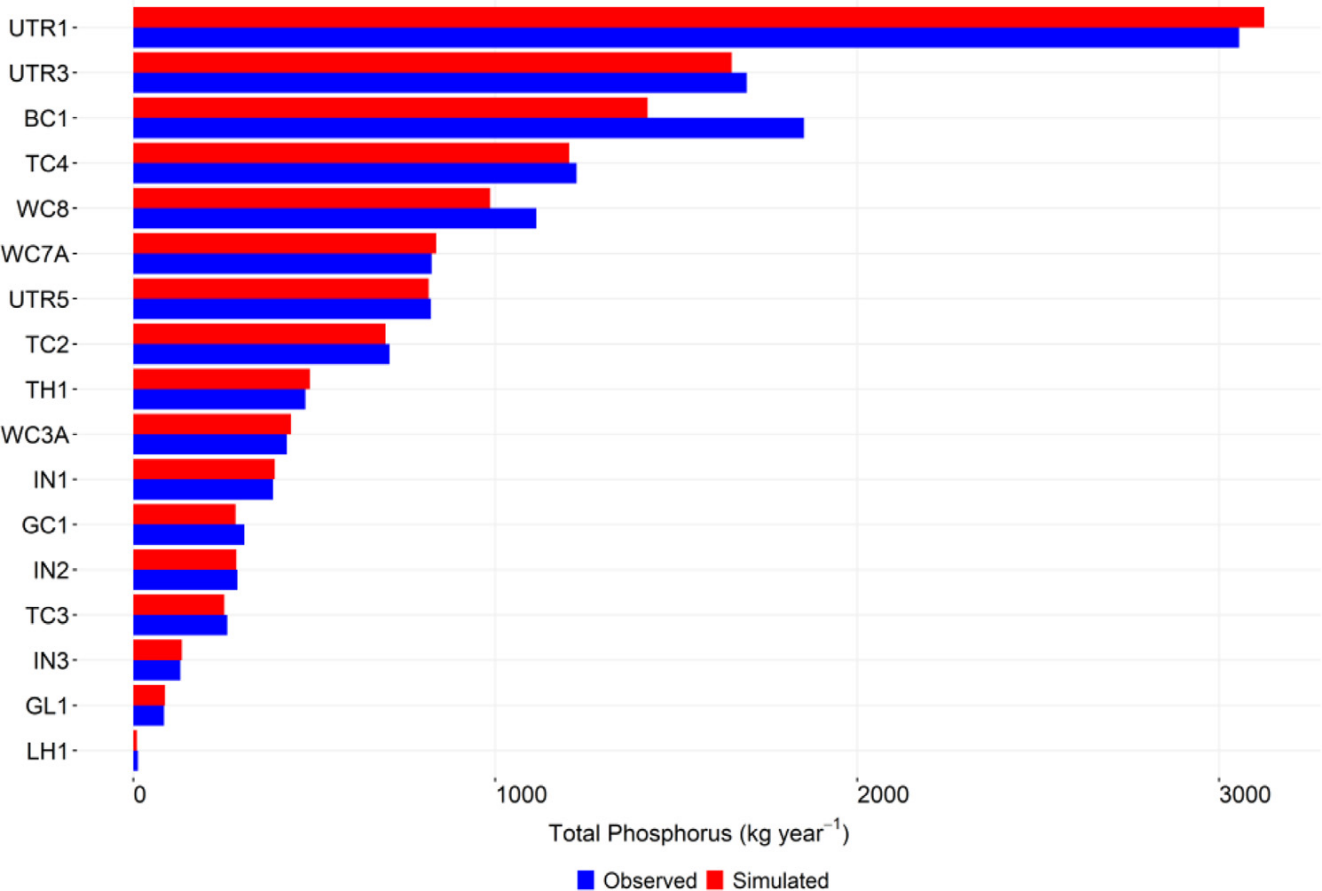
Simulated and observed total mean annual particulate phosphorus (PP) loads at watersheds from the Lake Tahoe basin in California/Nevada.

**Fig. 9:**
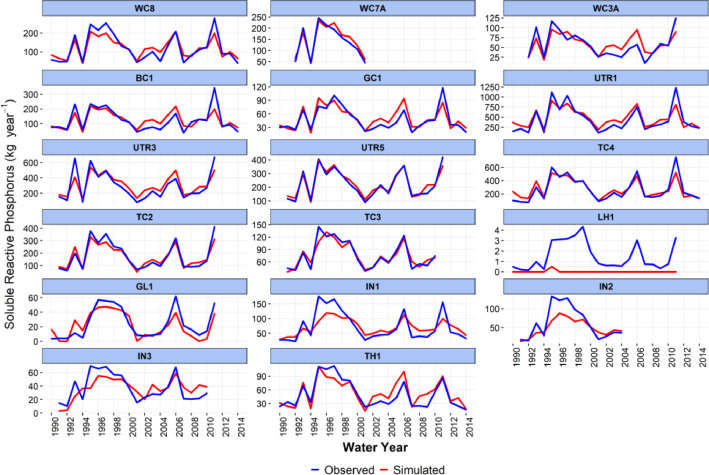
Simulated and observed total annual soluble reactive phosphorus (SRP) loads at watersheds from the Lake Tahoe basin in California/Nevada.

**Fig. 10:**
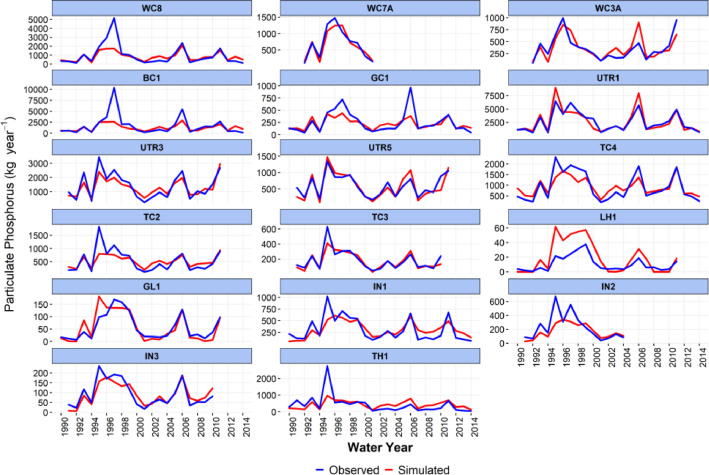
Simulated and observed total annual particulate phosphorus (PP) loads at watersheds from the Lake Tahoe basin in California/Nevada.

- **wepp/output** contains the main model outputs for each hillslope and for the watershed. Most of these files are self-explanatory, however, we encourage users to check the WEPP user manual [5] for additional information.

- **wepp/plots** contains maps of gridded soil loss following a flowpath run [6]

- **wepp/runs** contains all the main WEPP input files

- **nodb** filles, which are JSON serialized instances of wepppy.nodb classes used by WEPPcloud. These contain metadata related to the project. They are viewable in FireFox/Notepad++, etc.

**Fig. 11:**
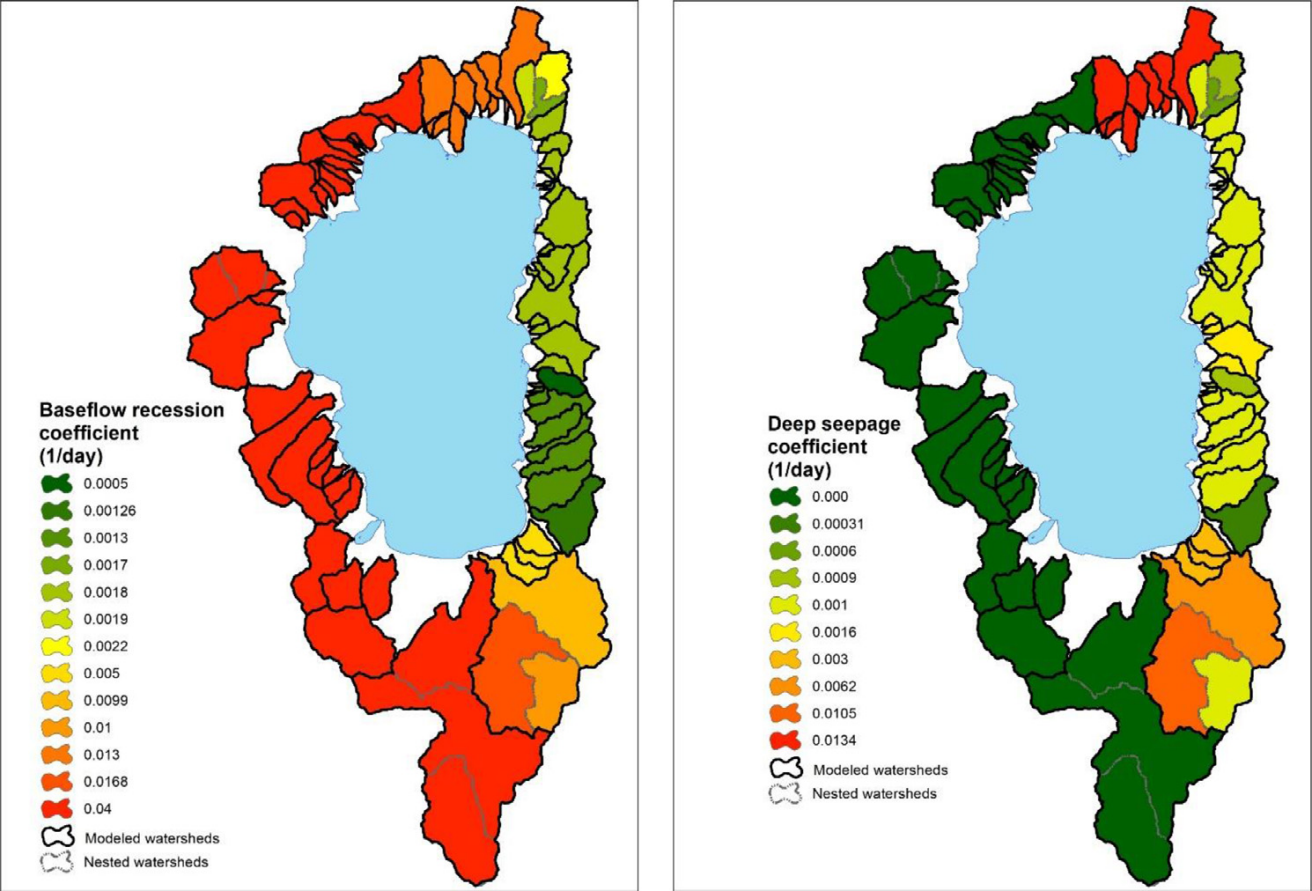
Interpolated estimated values of baseflow and deep seepage recession coefficients for the Lake Tahoe basin watersheds in California/Nevada.

**Fig. 12:**
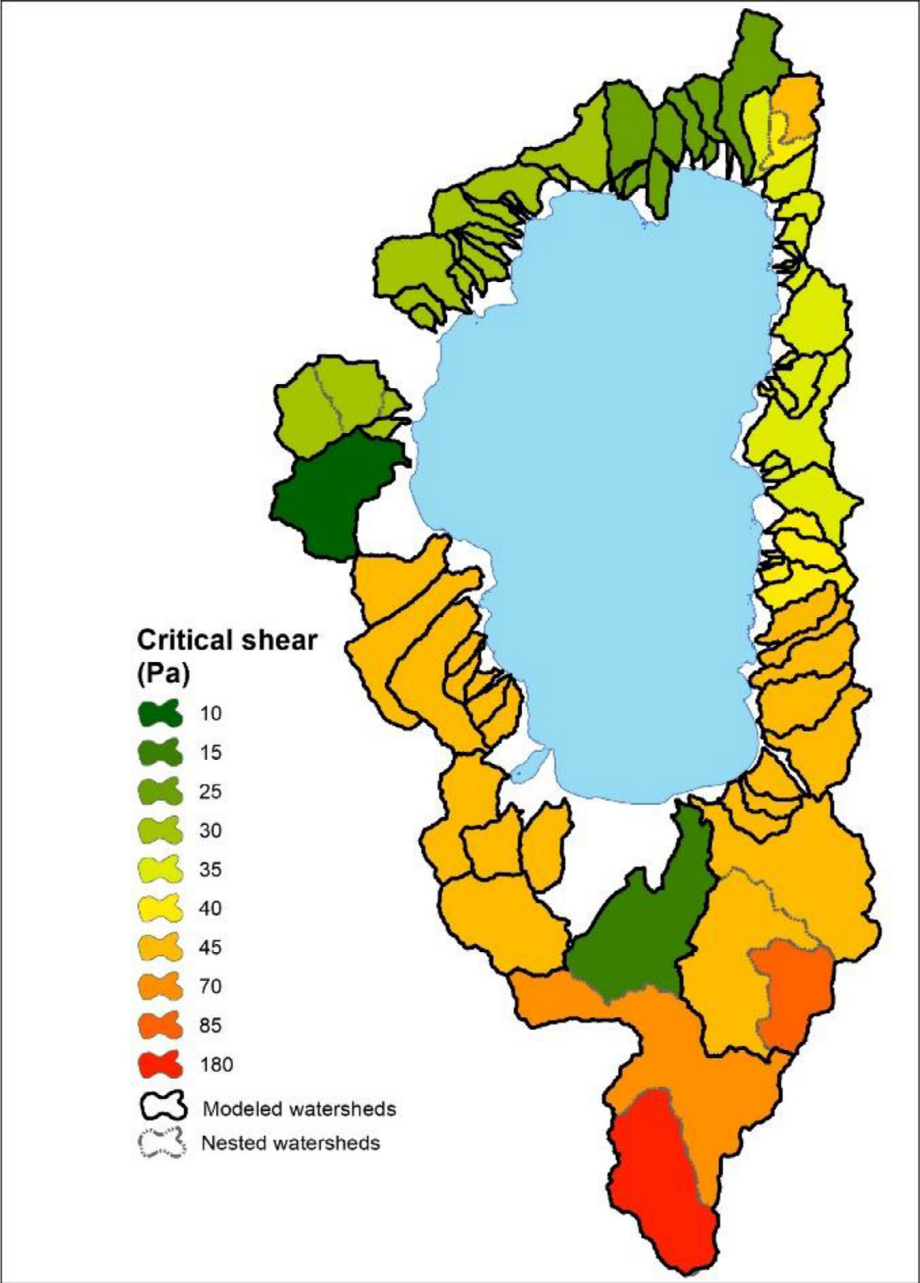
Interpolated channel critical shear for the Lake Tahoe basin watersheds in California/Nevada.

**Fig. 13:**
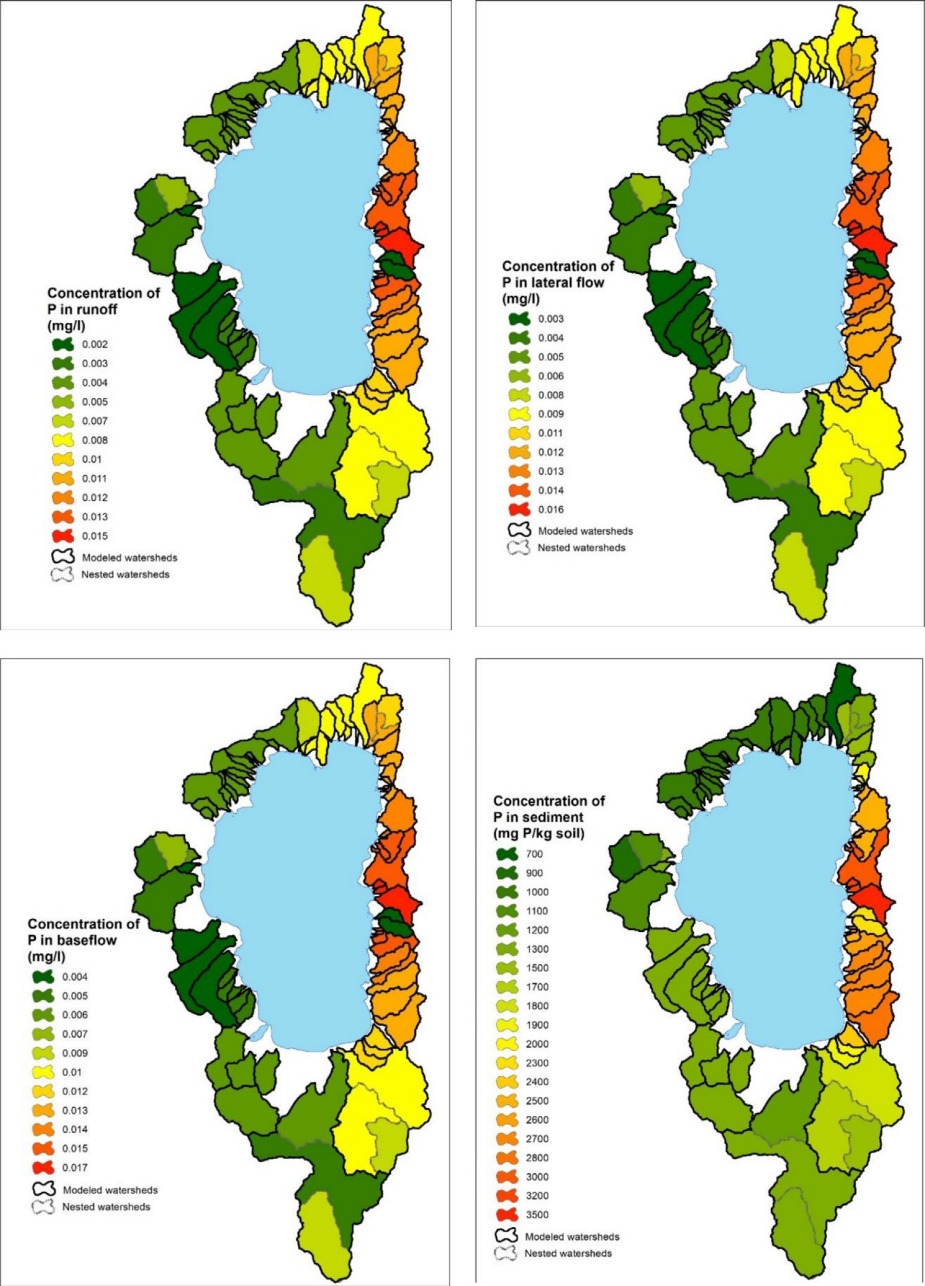
Interpolated phosphorus concentrations in runoff, lateral flow, baseflow and sediment from the Lake Tahoe basin watersheds in California/Nevada.

## 2 Experimental Design, Materials and Methods

**Fig. 14:**
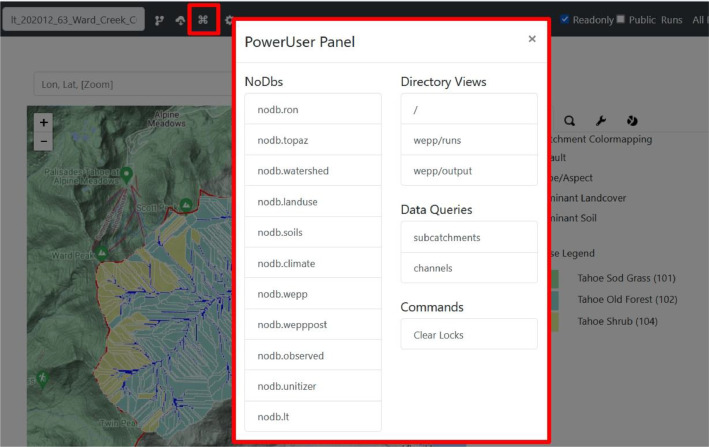
The PowerUser Panel for the Ward Creek Watershed, Lake Tahoe basin, California model run, which can be accessed at the following web weblink: https://wepp.cloud/weppcloud/runs/lt_202012_63_Ward_Creek_CurCond/cfg/

The hydrologic simulations were performed with the WEPPcloud interface [7,8] for 28 relatively undisturbed watersheds in the U.S. Pacific Northwest (Lake Tahoe basin, CA/NV; Bull Run Watershed, OR; Cedar River and Taylor Creek, WA, and two watersheds in Mica Creek Experimental Watershed, ID) and compared model outputs such as streamflow, sediment and phosphorus yield to observed data recorded at USGS gaging stations and recorded with flumes (Table 1; [1]). Each model run (including data input and output) can be viewed either online by accessing the web links in Table 1 or by accessing the zipped folders stored in the HydroShare repository. The WEPPcloud allows users to view most of the model input selections directly on the main page of the model run or in the PowerUser Panel (Fig. 14). The NoDbs folders contain model selections, while the wepp/runs and wepp/output folders contain all the input and output raw data files. The HydroShare repositories contain the same data in similar folders.

### 2.1 Model calibration

All model runs were performed initially with the WEPPcloud default parameters. We further minimally calibrated the model by downloading all the model input data, manually changing key calibrating parameters, and then rerunning the models with *wepppy-win-bootstrap* [9], a free Python package developed to facilitate model runs on Windows computers. Lastly, we reran the models on the WEPPcloud interface with the calibrating parameters. The calibration involved altering the linear baseflow recession coefficient (*k_b_* in /wepp/runs/gwecoeff.txt files), the saturated hydraulic conductivity of the underlying geology (*K_sub_* in /wepp/runs/[_].sol files), the rain/snow temperature threshold (T*_rain/snow_* in /wepp/runs/snow.txt file) for streamflow, channel bed critical shear stress (*τ_c_* in /wepp/runs/pw0.chn file) for sediment yield, and phosphorus concentrations in surface runoff, lateral flow, baseflow, and attached to sediment for phosphorus yield (in /wepp/runs/phosphorus.txt file). The minimal calibration was preferred to minimize potential issues with equifinality and to demonstrate model's predictive capabilities. Values for daily modeled streamflow at all watersheds and annual sediment and phosphorus yield at watersheds from the Lake Tahoe basin were compared to observed data (Fig. 2, Fig. 3, Fig. 4, Fig. 5, Fig. 6, Fig. 7, Fig. 8, Fig. 9, Fig. 10). Goodness-of-fit statistics (Nash-Sutcliffe Efficiency, the Kling-Gupta efficiency, and percent bias) and additional graphs can be found in [1].

### 2.1 Basin-scale model runs

In the Lake Tahoe Basin, we were interested in applying the WEPPcloud interface to all 63 watersheds that flow into the lake and further run the models for disturbed conditions (thinning, prescribed fire, wildfire, simulated fire) [1,2], however, the model calibration was performed only for 17 watersheds with long-term USGS data. Therefore, we manually distributed the calibrating parameters to the remaining watersheds based on the watersheds' similarities, parent material, and proximity (Fig. 11, Fig. 12, Fig. 13).

## CRediT Author Statement

**Mariana Dobre:** Conceptualization, Methodology, Data curation, Formal analysis, Visualization, Funding acquisition, Writing - Original Draft; **Anurag Srivastava:** Conceptualization, Methodology, Formal analysis, Software. **Roger Lew:** Conceptualization, Methodology, Software. **Chinmay Deval:** Data Curation, Visualization; **Erin S. Brooks:** Conceptualization, Methodology, Funding acquisition; **William J. Elliot:** Conceptualization, Methodology, Resources, Investigation, Funding acquisition, Writing - Review & Editing; **Peter R. Robichaud:** Conceptualization, Resources, Investigation, Funding acquisition, Writing - Review & Editing.

## Declaration of Competing Interest

The authors declare that they have no known competing financial interests or personal relationships that could have appeared to influence the work reported in this paper.

## Data Availability

Logan House (Original data) (HydroShare).
